# Encapsulation of Metal Nanoparticles within Metal–Organic Frameworks for the Reduction of Nitro Compounds

**DOI:** 10.3390/molecules24173050

**Published:** 2019-08-22

**Authors:** Sergio Navalón, Mercedes Álvaro, Amarajothi Dhakshinamoorthy, Hermenegildo García

**Affiliations:** 1 Departamento de Química, Universitat Politècnica de València, C/Camino de Vera, s/n, 46022 Valencia, Spain; 2 School of Chemistry, Madurai Kamaraj University, Madurai-625 021, India; 3 Instituto Universitario de Tecnologia Quimica (CSIC-UPV), Universitat Politecnica de Valencia, Av. De los Naranjos s/n, 46022 Valencia, Spain; 4 Center of Excellence for Advanced Materials Research, King Abdulaziz University, Jeddah 21589, Saudi Arabia

**Keywords:** metal–organic frameworks, metal nanoparticles, nitro compounds, reduction reaction

## Abstract

Nitro group reduction is a reaction of a considerable importance for the preparation of bulk chemicals and in organic synthesis. There are reports in the literature showing that incorporation of metal nanoparticles (MNPs) inside metal–organic frameworks (MOFs) is a suitable strategy to develop catalysts for these reactions. Some of the examples reported in the literature have shown activity data confirming the superior performance of MNPs inside MOFs. In the present review, the existing literature reports have been grouped depending on whether these MNPs correspond to a single metal or they are alloys. The final section of this review summarizes the state of the art and forecasts future developments in the field.

## 1. Introduction

**Figure 1:**
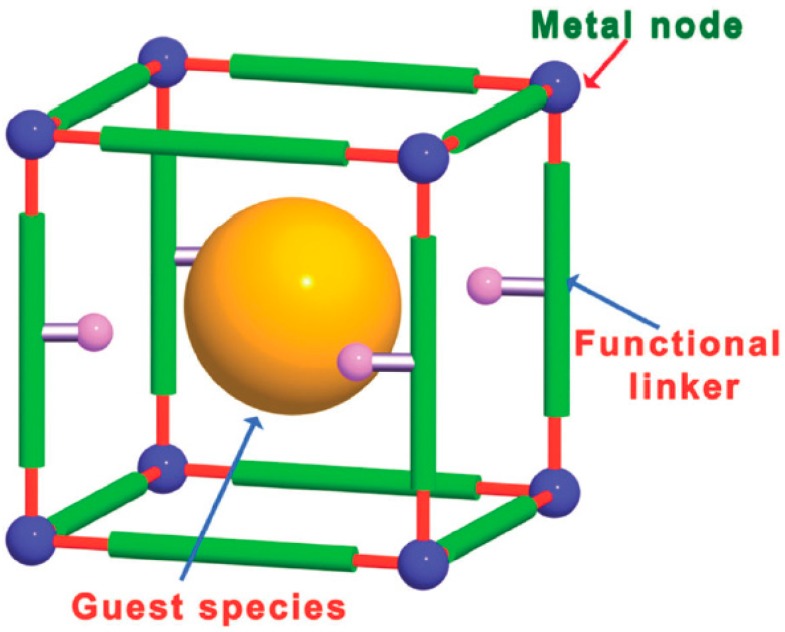
Possible location of active sites in MOFs, including metal nodes, functionalized organic linkers, and guest species such as MNPs in the pores. Reproduced with permission from [24].

Metal–organic frameworks (MOFs) are solid porous crystalline materials constituted by metallic nodes coordinated to rigid organic linkers, typically bi- or multipodal aromatic carboxylic acids or nitrogenated heterocycles [1,2,3,4,5,6,7,8,9]. MOFs are currently under intense investigation as solid catalysts, mainly due to the Lewis acidity of metal ions at the nodes [10,11,12,13,14,15], but also by possible acid [16] and basic [17] groups present at organic linker (Figure 1). Another possibility to use MOFs in heterogeneous catalysis is as support of metal nanoparticles (MNPs) and other types of guests that could act as active sites in catalysis [18,19,20,21,22,23,24,25,26,27,28,29,30,31,32].

For the last application, MOFs are especially suited materials because they exhibit high surface area, large porosity and the pore dimension can be tuned from micro to mesoporosity [3,4,5,33,34,35,36,37]. Of special interest for the present review is the case of noble MNPs hosted inside the pores of MOFs [20]. In this case, MOFs are used as supports and can stabilize the incorporated MNPs by providing a confined space that limits the growth of the MNPs by geometrical constraints [20,38,39,40]. These materials composed by MNPs incorporated inside MOFs (MNPs@MOFs) exhibit the intrinsic catalytic properties of the guest with some control in the accessibility to the site (shape selectivity) and some possible synergy by the presence of metal ions or functional groups at the linker that can cooperate to the catalysis [20,24]. 

The field of catalysis by MNPs@MOFs has been extensively reviewed [18,19,20,21,22,23,24,25,26,27,28,29,30,31], paying attention to various methodologies for preparation to ensure the internal location of the MNPs, characterization techniques and the advantages in catalysis that derive from the encapsulation of the MNPs inside MOFs. 

In the present review, we will focus on these types of solid catalysts for the specific reduction of nitro groups, particularly nitro aromatics. This reaction has considerable importance both from the academic and industrial points of view for the preparation of anilines as well as in organic synthesis combined with nitration of aromatic rings for the preparation of aniline derivatives [41,42,43].

The reduction of nitro groups to amino derivatives can be catalyzed by different metals depending on the nature of the reducing agent. As a general rule, the activity of these metals increases when they are prepared as MNPs. In this context, the activity and selectivity of MNPs for the specific case of nitro reduction also benefit from the incorporation of these MNPs inside MOFs.

One issue that has to be carefully addressed when using MOFs in catalysis is their structural stability. Although there are many MOFs that have limited thermal and chemical stability, there are by now several examples including various MIL (MIL: Materials Institute Lavoisier) [2,44,45] based materials and Zr^4+^-based MOFs [46,47] that have remarkable stability, both thermally and to chemical reagents. For these reasons as well as their large porosity and surface area, these types of structurally robust MOFs are the preferred hosts to incorporate MNPs.

Regarding the incorporation of MNPs inside MOFs, one of the general methodologies widely used is adsorption of a metal salt precursor over MOFs and their subsequent reduction by metal hydrides, hydrogen or other reducing agents to form MNPs@MOFs [18,20,21,28,31]. In this context, one procedure that is becoming widely used since it generally leads to internal location of the NPs is the so called “double solvent” method (DSM) in which the MOF is suspended in an apolar solvent such as hexane and the metal precursor is dissolved in a small volume of water corresponding to the internal porosity of the MOF [48,49]. This methodology relies on the hydrophilicity of the MOFs that prefers the adsorption of the water against apolar solvents.

Regarding the location of the MNPs, it is obvious that to be considered as located within the pores, the particle size of these MNPs should be smaller than the dimensions of the MOF cages and cavities [18,20,21,31,50]. Therefore, small particle size is a prerequisite to admit the incorporation of MNPs inside the pores. However, non-routine characterization techniques and particularly high resolution electron tomography are necessary to provide convincing evidence that the NPs are incorporated inside the pores [51]. Other measurements such as porosity and surface area and even shape selectivity are only indirect ways to address this issue. These indirect evidences assume that the decrease of porosity is due to the space occupied by MNPs. However, other possibilities such as pore blocking or location at the entrance of the pores could also result in decreased porosity of the materials or even in the selective formation of the product with smallest dimension without having MNPs inside the pores [18,20,21,28,31].

The as-prepared MNPs@MOFs should be characterized to provide evidence supporting the existence of MNPs within the pores of MOFs. Powder XRD of pristine and MNPs@MOFs must be compared to prove that the crystal structure of the MOF network is retained upon loading of metal MNPs. Further, gas adsorption measurements would provide data concerning surface area and pore volume before and after loading of MNPs. Brunauer–Emmett–Teller (BET) surface area and pore volume of MOF should become reduced upon loading of MNPs within the pores of MOFs, but a certain porosity should remain in MNPs@MOFs to exhibit efficient catalytic activity. In fact, a decrease in the BET surface area is widely considered as one of the stronger evidences to confirm the internal localization of MNPs within MOFs pores. X-ray photoelectron spectroscopy (XPS) reports on the oxidation state of the MNPs loaded over MOFs. On other hand, transmission electron microscope (TEM) images show information about the particle size distribution of MNPs inside the pores of MOFs. TEM studies should be performed carefully to avoid damage of MOF matrix due to high energy electron beam required in high resolution imaging. It is particularly important to characterize by TEM the reused MNP@MOFs catalyst to ensure the retainment of particle size upon repeated cycles. Comparison of the particle size between fresh and reused solid catalysts must be provided as an evidence of catalyst stability. Scanning electron microscope (SEM) images show the structural morphology of MOFs before and after loading of MNPs and after activity tests. In general, the as-prepared hybrid solid must be characterized before and after catalytic reactions by any of these techniques in order to ascertain the stability of MNPs during the course of a catalytic reaction.

The present review has been organized in two main sections. One of them describes the use of single MNPs encapsulated inside MOFs as catalysts for nitro group reduction. The subsequent section describes examples in where two metals forming alloys or even one example of a trimetallic alloy have been prepared inside the MOFs and used as catalysts. In these examples, the preparation procedure and the catalytic activity will be described, paying attention to show the superior catalytic performance of these MNPs@MOFs composites in respect to other supported MNPs or homogeneous catalysts. Reusability and catalyst stability is also sufficiently stressed. The final section provides a brief summary of the current state of the art and the outlook for future development of the field.

## 2. Monometallic MNPs@MOFs

In this section, the use of monometallic MNPs supported on MOFs to effect the reduction of nitro compounds will be described. Besides molecular H_2_, other reagents, such as formate and metal hydrides have been used, each requiring a different metal catalyst. Table 1 summarizes the list of various MNPs@MOFs as heterogeneous solid catalysts, particle size of MNPs, reducing agents and corresponding catalytic reactions.

**Figure 2:**
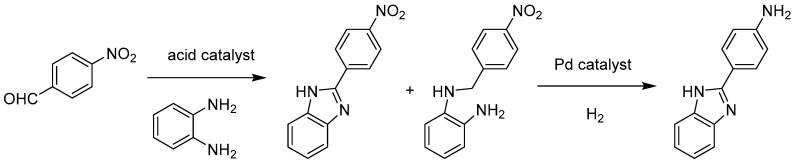
Synthesis of 2-(4-aminophenyl)-1*H*-benzimidazole via tandem catalysis involving acid catalysis and catalytic hydrogenation of nitro groups using Pd@MIL-101(Cr) as bifunctional catalyst.

Traditionally, hydrogenation reactions have been promoted by using noble MNPs such as Pd or Pt dispersed in a high surface area support. In this context, the higher surface area and porosity of MOFs offer several advantages for the deposition of MNPs in its framework. One of the frequently studied MOFs in catalysis is the MIL-101 MOFs due to their robust structure under reaction conditions. The MIL-101(Cr) framework, Cr_3_X(H_2_O)_2_O(BDC)_3_·nH_2_O (BDC = benzene-1,4-dicarboxylate, X = F, OH, n ≈ 25) exhibits a highly-stable three-dimensional network with two types of giant cages with diameters of 2.9 and 3.4 nm accessed through smaller pentagonal (2.9 nm) and hexagonal (3.4 nm) windows and large BET surface area (around 3600 m^2^/g). Hence, Pd NPs have been incorporated within MIL-101(Cr) (Pd@MIL-101(Cr)) using DSM and are stabilized by van der Waals interactions. The activity of Pd@MIL-101(Cr) was tested in a tandem reaction involving Lewis acidity from unsaturated metal centers of MIL-101 and encapsulated Pd NPs as hydrogenation sites [52]. TEM images showed that the Pd NPs in Pd@MIL-101(Cr) are highly dispersed with the mean diameters of 2.5 ± 0.3 nm. This average particle is compatible with these Pd NPs being located within the pores of MIL-101 since they are smaller than the pore size. The catalytic performance of Pd@MIL-101(Cr) was tested in the synthesis of 2-(4-aminophenyl)-1H-benzimidazole by cyclocondensation of o-phenylenediamine and 4-nitrobenzaldehyde via tandem reaction involving acid catalysis and catalytic hydrogenation steps (Figure 2). A complete conversion of 4-nitrobenzaldehyde was achieved with Pd@MIL-101(Cr), reaching >99% yield of the final desired product after tandem catalysis. In contrast, homogeneous catalysts such as palladium acetate together with CuI gave lower yields around 80% and, in addition, this homogeneous catalytic system requires a tedious purification procedure [66]. A control experiment in the absence of catalyst gave only 30% conversion to an intermediate product of the tandem reaction. Also, commercial Pd/C catalyst resulted in 50% conversion to afford the intermediate product corresponding to the first step of the tandem process and the reaction could not proceed further due to the lack of acidity. In another control experiment with MIL-101(Cr), the reaction was terminated by affording intermediate product due to the lack of Pd NPs. Furthermore, reusability tests using Pd@MIL-101(Cr) indicated that the activity and selectivity remained stable for three cycles. TEM images of the reused Pd@MIL-101(Cr) have shown that the sizes of Pd NPs are still retained after three runs. These results clearly illustrate the synergistic effect between Lewis acidity provided by the MIL-101(Cr) metal nodes and the confinement effect of MIL-101(Cr) lattice embedding Pd NPs to reach superior activity of the Pd@MIL-101(Cr) composite in the synthesis of heterocyclic compounds.

**Figure 3:**
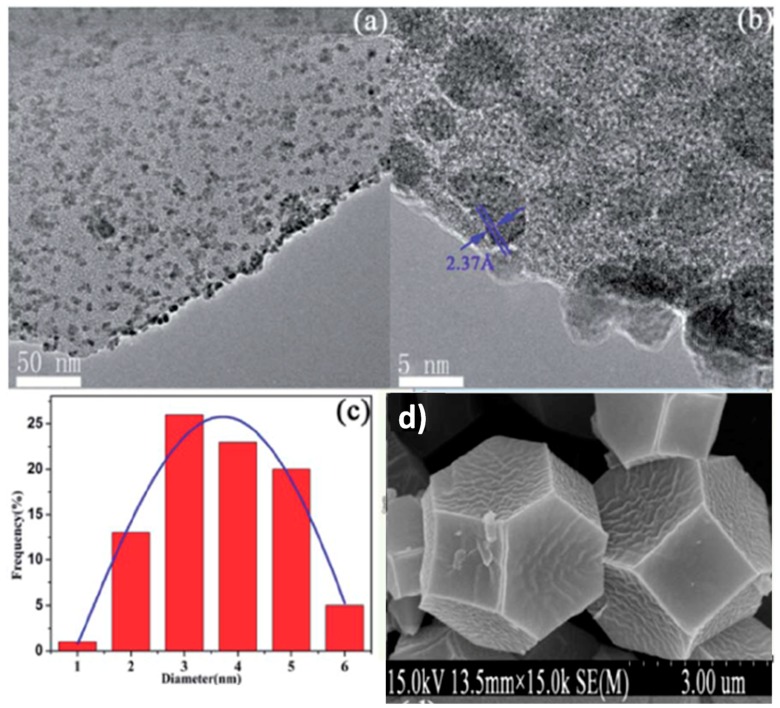
TEM images (**a**,**b**) and Pd NPs size distribution (**c**) for Pd(0.5wt%)/DUT-67. (**d**) SEM image of DUT-67. Reproduced with permission from [53].

Zirconium-based MOFs frequently exhibit high thermal and chemical stability together with porosity and, therefore, are good candidates for their use as catalysts. In one study, Pd NPs supported in DUT-67(Zr) were employed as catalysts for the hydrogenation of nitrobenzene under mild reaction conditions [53]. DUT-67(Zr) is a MOF constituted by Zr_6_O_4_(OH)_4_ metal nodes connected by thiophenedicarboxylic acid leading to the formation of a porous 3D structure with one cuboctahedra cage (1.16 nm) and another octahedral (1.16 nm) cavity. Pd NPs supported in DUT-67(Zr) were prepared by mixing PdCl_2_ as precursor with previously formed DUT-67 both in DMF and, then, adding NaBH_4_ as reducing agent leading to the formation of Pd NPs. This methodology, however, resulted in the formation of Pd NPs with particle sizes (3.5 nm average, Figure 3) larger than the MOF cages. Thus, Pd NPs are mostly located on the crystal planes of the MOF with a small proportion inside the porous matrix, but they are stabilized by van der Waals forces. The Pd(0.5 wt%)-DUT-67 catalyst was able to promote the selective hydrogenation of nitrobenzene to aniline using ethanol as solvent at 60 °C, while maintaining its stability as revealed by powder X-ray diffraction (PXRD). Regardless, the ability of the Pd-DUT-67 material to act as hydrogenation catalyst indicates that further efforts should be done to prepare a material with all Pd NPs encapsulated inside the MOF lattice with the aim of increasing their overall catalytic activity and, particularly, the stability of encapsulated MNPs.

**Figure 4:**
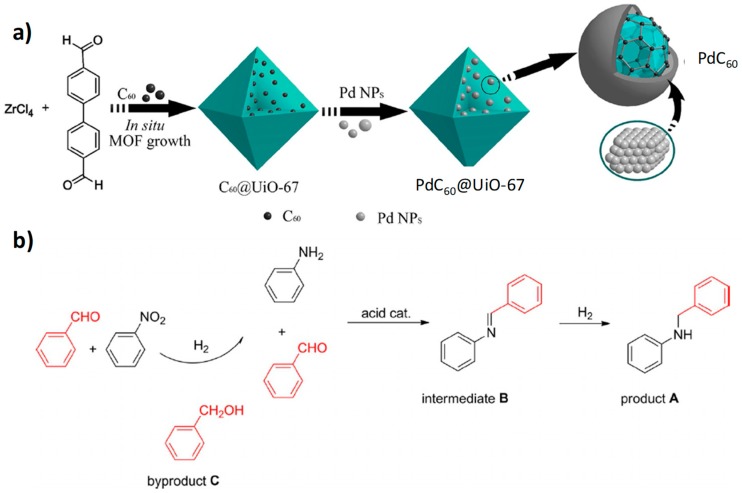
(**a**) Schematic illustration for the sequential synthesis of C_60_@UiO-67 and PdC_60_@UiO-67. (**b**) Synthesis of secondary N-benzylanilines through hydrogenation of nitrobenzene and reductive amination of benzaldehyde. Reproduced with permission from [54].

More recently, Pd NPs coordinated to fullerene C_60_ have been incorporated in the large pores of UiO-67(Zr) (1.2 to 2.3 nm; 1575 m^2^ g^−1^) and, then, employed as a multifunctional heterogeneous catalyst for the synthesis of secondary arylamines via tandem hydrogenation reaction of nitrobenzene and reductive amination of benzaldehyde (Figure 4) [54]. UiO-67(Zr) is constituted by 4,4′-biphenyldicarboxylate ligands coordinated to Zr_6_O_4_(OH)_4_ clusters. Previous studies have reported the use of C_60_ molecules to support MNPs [67], such as PdC_60_ [68,69] for hydrogenation of nitro compounds and acetylene derivatives. Other studies have also shown that C_60_ alone can be employed as catalyst for the photocatalytic hydrogenation at room temperature of aromatic nitro compounds to their corresponding amino derivatives at 1 bar H_2_ [70]. The PdC_60_@UiO-67(Zr) (~1 wt% Pd and ~19 wt% C_60_) was characterized by the presence of homogeneously dispersed Pd NPs of 5 ± 2 nm size [50]. Regardless the attribution of the decrease of the measured surface area and pore volume in the series of UiO-67(Zr) (1739 m^2^ g^−1^, 0.99 cm^3^ g^−1^), C_60_@UiO-67(Zr) (1488 m^2^ g^−1^, 0.58 cm^3^ g^−1^), PdC_60_@UiO-67(Zr) (506 m^2^ g^−1^, 0.35 cm^3^ g^−1^) to the internal location of C_60_ and/or C_60_Pd counterparts, it is clear that the size of some Pd NPs larger than the diameter of the UiO-67(Zr) cavities should result in their partial location on the outer part of the MOF particles. It has to be considered that external deposition of Pd NPs on the UiO-67 crystallites can also result in a decrease of the N_2_ adsorption by partial blocking of the pore entrances. XPS measurements of the PdC_60_@UiO-67(Zr) and the reference PdC_60_ material showed a shift of the peaks corresponding to Pd to higher binding energies in respect to those of Pd NPs supported on UiO-67(Zr) sample. This observation was attributed to the partial charge transfer from Pd NPs coordinated to C_60_. The activity of PdC_60_@UiO-67(Zr) was higher in terms of conversion (100%) and selectivity (76% to *N*-benzylaniline, product A in Figure 4; and 24% to benzyl alcohol, byproduct C in Figure 4) in respect to the use of PdC_60_ or Pd/C as reference catalysts. The higher activity of PdC_60_ incorporated within UiO-67(Zr) is probably due to the Lewis acidity of the metal nodes, even though they are not strong, and the coordination of Pd NPs with C_60_. The low activity obtained with the use of Pd@UiO-67(Zr) or a physical-mixture of C_60_Pd and UiO-67(Zr), indirectly demonstrates the cooperative effects of the different components in C_60_Pd@UiO-67(Zr). The catalyst was reused five times without significant decrease in its catalytic activity while maintaining its initial structure as revealed by chemical analysis of the used catalyst, powder XRD, HRTEM and isothermal N_2_ adsorption measurements.

In the last decade several authors have proposed the use of formic acid as a green and sustainable hydrogen source, as an alternative in some applications to molecular H_2_. Some of the advantages of the use of formic acid or its derivatives such as ammonium formate or sodium formate in respect to H_2_ include their easy storage, transportation and manipulation. In this context, ruthenium NPs supported on the robust UiO-66(Zr) material have shown to be an appropriate catalyst for the reduction of nitroarenes to their corresponding amines using formic acid as a hydrogen source [55]. Small ruthenium NPs (1.07 nm) were incorporated into the cavities of UiO-66(Zr) (pore sizes from 0.6 to 1 nm) [71] by adsorption of RuCl_3_ in UiO-66(Zr) using ethyl acetate as solvent and, then, reduction of Ru^3+^ to Ru^0^ using NaBH_4_ [55]. Isothermal N_2_ adsorption measurements confirm the decrease of BET and pore volume values of Ru-UiO-66(Zr) (876 m^2^ g^−1^, 0.39 cm^3^ g^−1^) in respect to the parent UiO-66(Zr) (1276 m^2^ g^−1^, 0.53 cm^3^ g^−1^), presumably due to the location of Ru NPs into the pores of UiO-66(Zr) solid. XPS measurements evidence the co-existence of Ru^0^, RuO_2_ and RuO_2_.xH_2_O in the Ru-UiO-66(Zr) material. The Ru-UiO-66(Zr) hybrid stabilized by van der Waals forces was employed as catalyst for the complete nitrobenzene reduction to aniline by formic acid under optimized reaction conditions. The benefits of water in the reaction medium were ascribed to dissociation of formic acid into H^+^ and HCOO^−^. Interestingly, the 2-propanol and H_2_O mixture (9:1 volume ratio) is close to its azeotropic composition and, therefore, it allows easy solvent recycling. The heterogeneity of the reaction was confirmed by hot-filtration test. The catalyst can be reused six times without observing decrease of catalytic activity. Characterization of the used catalyst by TEM revealed a slight increase of the ruthenium particle size in respect to the fresh sample (1.27 vs. 1.07 nm), while XPS of the used sample confirmed that the oxidation state of ruthenium NPs is almost the same as that of the fresh sample. Also, inductively coupled plasma atomic emission spectrometry (ICP-AES) revealed the almost complete absence of metal leaching during the reaction. The scope of the reaction was studied using a series of nitrobenzene compounds substituted with electron donating or withdrawing groups, observing in all cases quantitative conversions with complete selectivities, except in the case of *p*-nitrobenzaldehyde or nitrocyclohexane where the formation of oligomers or unidentified compounds was observed.

**Figure 5:**
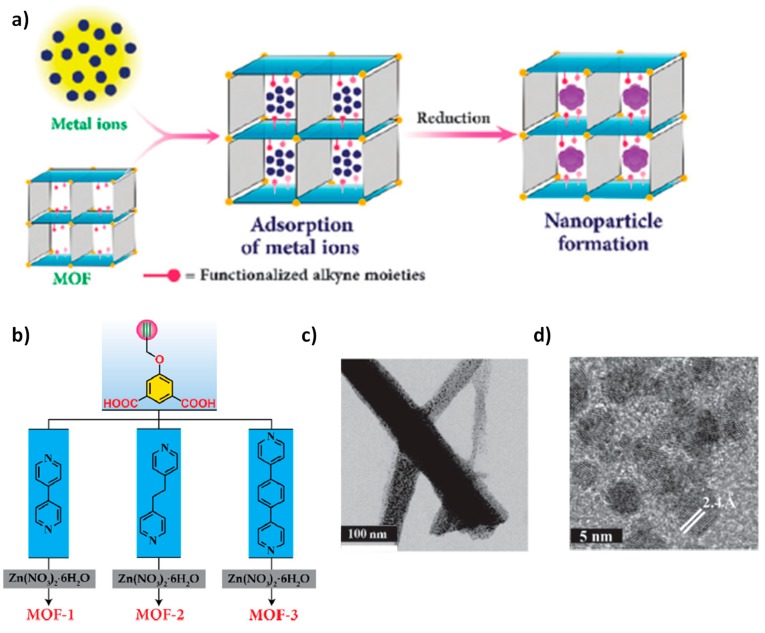
(**a**) Schematic illustration of MNPs synthesis on ethynyl-based MOFs. (**b**) Chemical structure of the ligands employed for the preparation of the MOFs, (**c**) STEM bright field image of the Au@MOF-3. (**d**) HRTEM image showing lattice spacing (d = 2.4 Å) corresponding to (111) plane of fcc Au. Reproduced with permission from [56].

Metal hydrides have been traditionally employed as reducing agents. In the last few decades, stable and easily-handled hydrides such as NaBH_4_ have been employed together with a catalyst to promote reduction reactions. In this line, MNPs supported on MOFs have been employed as heterogeneous catalysts for the reduction of aromatic nitro compounds to their corresponding aromatic amines using NaBH_4_ as reducing agent. One of the challenges in the area of heterogeneous catalysis using MOFs together with MNPs is to develop reliable procedures for the preparation of well-dispersed small MNPs encapsulated into the framework cavities [18,20,21,22,26,29,31,72]. In general, one of the important parameters to control the MNP size distribution is the metal loading. The use of low metal loadings allows obtaining small MNPs (<5 nm) with high catalytic activity. However, as the metal loading into the MOF increases beyond 20 wt% the average particle size and standard deviation of the loaded MNPs increase considerably resulting in a catalyst with decreased catalytic activity. Particularly, a decrease in the values of turn over numbers (TON) and frequencies (TOF) is observed upon metal loading increase [38]. In this sense, several studies have focused on developing new methodologies to increase the metal loading into the MOF cavities, while preserving the internal location of the NPs and, therefore, maintaining small particle size of the MNPs. In one example, exceptionally high loading of Au NPs into the cavities of three MOFs functionalized with alkyne moieties has been achieved [56]. The 3D MOFs were prepared by solvothermal conditions using 5-(prop-2-yn-1-yloxy)isophthalic acid and 4,4′-bipyridyl derivatives as organic ligands and Zn(NO_3_)_2_ as the metal source (Figure 5). This strategy takes advantage of the high alkynophilicity of Au^3+^ ions that allows the high dispersion of this ion in the interior of the material and, as consequence, the resulting Au NPs into the MOF cavities as small NPs (1.85 ± 0.85 nm)) at high metal loadings (~50 wt%). To further validate the role of the ethynyl groups into the MOF structure for the formation of small Au NPs at high loading, an analogous MOF functionalized with phenyl moiety, instead of the ethynyl group was also prepared as a control. In this case, a gold metal loading of 5 wt% resulted in the formation of gold agglomerates with particle sizes larger than 12 nm located on the outer surface of the MOF particles. Au NPs supported on the ethynyl-based MOF by electrostatic interaction resulted in being a stable and reusable catalyst to perform the reduction of 4-nitrophenol or 2,4-dinitrophenol to their corresponding amino derivatives by using NaBH_4_ in aqueous media. The activity of this catalyst for the 4-nitrophenol reduction by NaBH_4_ was more than one order of magnitude (rate constant 6.64 × 10^−2^ s^−1^) greater or comparable than analogous Au catalysts supported on Nano ActiveTM Mg [59], meso-HAP (HAP: hydroxyapatite) [73], PAMAM G3 (PAMAM G3: 3^rd^ generation poly(amidoamine)) [74], SiO_2_ [75], SiO_2_@Yne (Yne: alkynyl carbamate moieties) [76], Au-SiO_2_@AeThio (AeThio: amino-sulfide branches) [77], CeO_2_-NT (NT: nanotubes) [78], PPy-NTs (PPy: polypyrrole) [79], MIL-100(Fe) [80], or hm-ZrO_2_ (hm: hollow-mesoporous) [81]. Interestingly, if the reduction of 4-nitrophenol takes place using ethanol as solvent the resulting product was its azo derivative. It was proposed that ethanol as solvent favors the accumulation of nitroso intermediate that reacts further with the amino product leading to the formation of the azo compound. The scope of the azo compound formation was validated for a series of nitrobenzene derivatives containing electron donor or withdrawing groups.

**Figure 6:**
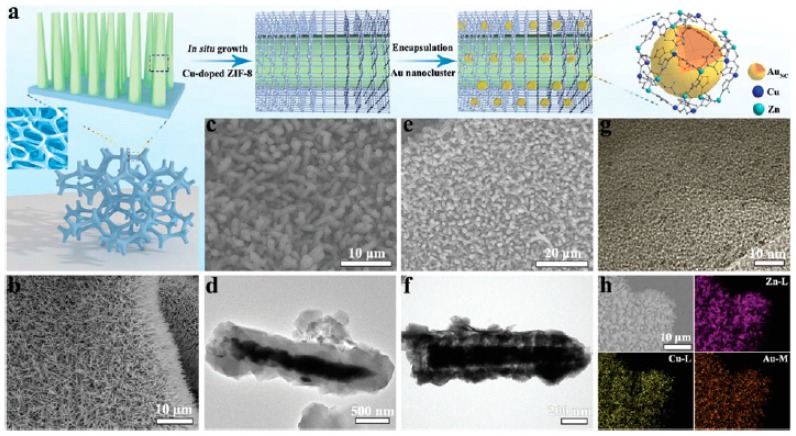
(**a**) Schematic diagram illustrating the synthetic procedures of the catalyst samples. (**b**) The SEM image of ZnO NRs. (**c**) The SEM image of ZIF-8(Cu) nanorod arrays (NRAs). (**d**) The TEM image of ZIF-8(Cu) NRAs. (**e**) The SEM image of AuNC@ZIF-8(Cu) NRAs (NC: nanoclusters). (**f**) The TEM image of AuNC@ZIF-8(Cu) NRAs. (**g**) The HRTEM image of AuNC@ZIF-8(Cu) NRAs. (**h**) The element mapping of Zn, Cu and Au of AuNC@ZIF-8(Cu) NRAs. Reproduced with permission from [57].

In addition to the importance of obtaining high metal loading with small particle size, the electronic structure of MNPs can determine its catalytic activity. Recently, gold nanoclusters (3 wt%, <2 nm) encapsulated in a Cu-doped ZIF-8 nanorod arrays (Au@ZIF-8(Zn,Cu), 0.7 and 1.2 nm pore diameters) on supported Ni foam (Figure 6) have been employed as catalyst with enhanced activity to promote the 4-nitrophenol reduction to 4-aminophenol using NaBH_4_ as reducing agent [57]. It has been proposed that doping of Cu^2+^ ions in the ZIF-8(Zn) framework modifies the electronic structure of encapsulated gold nanoclusters favoring the formation of gold hydride intermediates and, in this way, Cu^2+^ increases indirectly the catalytic activity of Au NPs. XPS measurements allow the characterization of Au^0^ nanoclusters with a positive 0.4 eV shift in the binding energy of the Au 4f peak in respect to the standard value of metallic Au NPs. The observation of the electropositivity of Au nanoclusters in Au@ZIF-8(Zn,Cu) was attributed to their interaction with electrophilic coordinatively unsaturated Cu N_x_ (x < 4) ions. In support of this proposal, the Cu K-edge in synchrotron X-ray absorption near-edge structure (XANES) of Au@ZIF-8(Cu) confirmed the presence of Cu^2+^ ions with a slight electronegativity. Fourier-transformed k_3_-weighted extended XAFS (FT-EXAFS) confirms the presence of coordinatively unsaturated Cu–N sites (3.5 coordination number) in the Au@ZIF-8(Cu) solid, while Au L_3_-edge in XANES reveals the presence of Au–Cu coordination in the first shell of gold centers. The Au@ZIF-8(Zn,Cu) material exhibited higher catalytic activity than that of either Au@ZIF-8(Zn), unsupported Au NPs, ZIF-8(Zn,Cu) or ZnO. Importantly, the higher the Cu^2+^ doping in Au@ZIF-8(Cu), the higher the catalytic activity. The catalyst was reused for ten times without observing decrease in its catalytic activity and maintaining its structure according to SEM, TEM and XPS analyses. The scope of the catalyst was confirmed by performing the catalytic reduction of *o*- and *m*-nitrophenol as well as several nitrobenzene derivatives having electron donor or electron withdrawing substituents to their corresponding amino derivatives.

**Figure 7:**
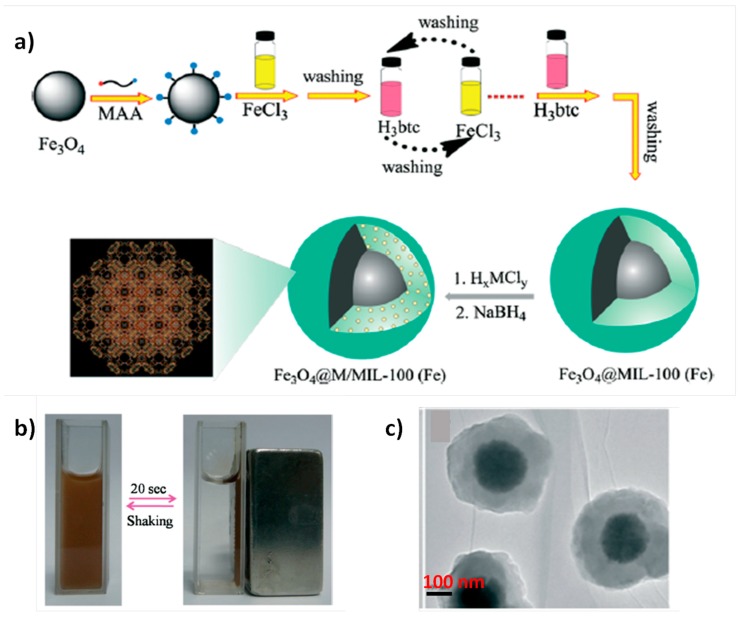
(**a**) Route followed for the fabrication of Fe_3_O_4_@M/MIL-100 (Fe) (M = Au, Pt, Pd) microspheres. MAA, mercaptoacetic acid; H_3_btc, 1,3,5-benzenetricarboxylic acid. (**b**) Magnetic separation–redispersion process of Fe_3_O_4_@Pt/MIL-100(Fe) microspheres. (**c**) TEM images of individual Fe_3_O_4_@MIL-100 (Fe) core–shell nanospheres after 60 assembly cycles. Reproduced with permission from [58].

Catalyst recovery and recycling is one of the important issues in the development of heterogeneous catalysts for industrial applications. Traditionally, filtration or centrifugation are the most widely used methodologies for the separation of the catalyst from batch reactors. Other possibility consists in the preparation of a magnetic catalyst that can be easily recovered by applying weak magnetic fields. In one example of this strategy, a core–shell material composed by magnetite NPs (core) surrounded by MIL-100(Fe) solid (shell) loaded with noble NPs has been prepared and employed as magnetically recoverable catalyst to effect the reduction of aromatic nitro compounds to their corresponding amines by using NaBH_4_ as reducing agent [58]. Figure 7 shows the preparation of the catalyst by obtaining in a first step the Fe_3_O_4_ core (~250 nm) functionalized with mercaptoacetic acid (MAA) and, then, a MIL-100(Fe) shell was prepared via layer-by-layer assembly process that allows to control the shell thickness from about 25 to 100 nm. By means of the deposition-reduction method with NaBH_4_, Au (2.0 ± 0.2 nm), Pd (2.2 ± 0.2 nm) or Pt (1.9 ± 0.2 nm) NPs were incorporated within the mesopores of the MIL-100(Fe) shell in the Fe_3_O_4_@MIL-100(Fe) composite. Fe_3_O_4_@MIL-100(Fe)-Pt material exhibited the highest activity for 4-nitrophenol reduction to 4-nitroaniline by NaBH_4_ with an apparent first-order rate constant of 2.58 min^−1^. This value is higher than those previously reported for other noble metal-based catalysts such as Au_core_Ag_shell_-ZIF-8 [82], MIL-100(Fe) [80], or magnetic double-shell Fe_3_O_4_@TiO_2_/Au@Pd@TiO_2_ microsphere (Fe_3_O_4_ core and double TiO_2_ shells with Au and Pd NPs) [83]. The scope of the reaction was studied by reducing a variety of nitrophenol derivatives (TOF 450–3573 h^−1^), nitroaniline compounds (1297–2091 h^−1^) and *p*-nitrophenylhydrazine (TOF 563 h^−1^). The most active Fe_3_O_4_@MIL-100(Fe)-Pt catalyst was reused ten consecutive times without loss of the catalytic activity. It should be noted that after each catalytic reaction the catalyst recovery was done using a magnet and, then, washed with ethanol and dried before a new catalytic cycle. ICP-AES analysis of the liquid phase did not detect the presence of Pt, while nearly 80% of the initial Pt content was maintained in Fe_3_O_4_@MIL-100(Fe)-Pt after ten cycles.

**Figure 8:**
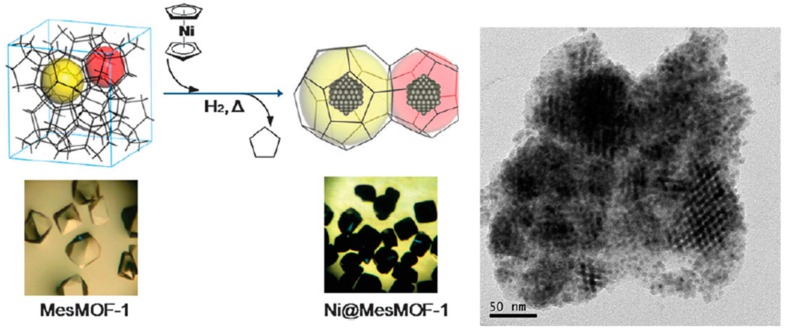
Two-step procedure for the preparation of Ni@Mesoporous-MOF-1. The pictures for the mesoporous MOF crystals before and after Ni embedment. The rightmost panel shows a TEM image of mesoporous MOF (30 wt%) where lattice fringes are observed, indicating Ni NPs are aligned with a long range order. Reproduced with permission from [59].

MNPs of base transition metals such as nickel or iron can be employed as cost-effective alternatives to noble metals to promote NaBH_4_ activation in the reduction of nitro aromatics. In one example, Ni NPs encapsulated into the cavities of a mesoporous MOF have been prepared and tested for the nitrobenzene reduction using NaBH_4_ [59]. With the aim to avoid MNP agglomeration on the crystal surface, Ni NPs have been immobilized into the MOF framework by gas-phase adsorption and, then, a reduction step using H_2_ (Figure 8). The mesoporous MOF used in the study is constituted by triazine-1,3,5-tribenzoate organic ligands connected to terbium ions leading to the formation of a 3D structure with two types of mesocages (3.9 and 4.7 nm in diameter), connected through pentagonal and hexagonal windows of 1.5 and 1.7 nm diameters, respectively. Interestingly, it was possible to achieve the incorporation of Ni NPs into the mesocages of the MOF at loadings as high as 20, 30 and 35 wt% without compromising the particle size that remained between 1.4 to 1.9 nm. The Ni NPs are well-dispersed and uniformly distributed without observation of random aggregates. The Ni-MOF material was employed as a reusable heterogeneous catalyst (three uses) for the complete conversion with full selectivity of nitrobenzene to aniline in methanol as solvent. The higher activity obtained using the Ni-MOF at 20 wt% loading in respect to that at 35 wt% was attributed to the higher surface area and pore volume of the former (470 m^2^/g and 0.28 cm^3^/g vs. 300 m^2^/g and 0.17 cm^3^/g).

## 3. Bimetallic and Trimetallic MNPs@MOFs

**Figure 9:**
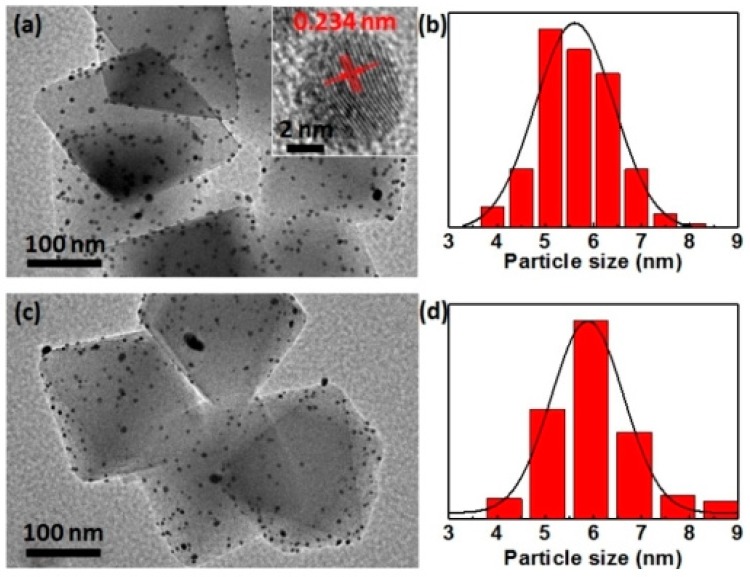
TEM images and corresponding histograms of particle size distribution for as-synthesized Au-Pd_0.03_@UiO-66-NH_2_ (**a**,**b**) and the sample after reaction (**c**,**d**). Reproduced with permission from [60].

Alloying two or more different transition metals allows the catalytic activity of the MNPs to be tuned. In one of the examples, alloyed Au–Pd NPs were loaded into UiO-66-NH_2_ MOF catalyst via adsorption/reduction method to obtain AuPd@UiO-66(Zr)-NH_2_ and their activity was examined in the reductive amination with nitroarenes [60]. Powder XRD indicated no changes in the crystallinity of UiO-66(Zr)-NH_2_ during the loading of these NPs. TEM images clearly showed that the average particle diameter of Au-Pd_0.03_ in Au-Pd_0.03_@UiO-66(Zr)-NH_2_ was 5.3 nm and further Pd and Au NPs existed with uniform dispersion and nearly consistent distribution of bright spots (Figure 9). The presence of accessible Pd atoms on the sample of the alloy NPs was revealed by IR spectroscopy using CO as a probe molecule. The catalytic performance of Au-Pdx@UiO-66(Zr)-NH_2_ was studied in the reductive amination of nitrobenzene to *N*-phenylbenzylamine. Au-Pd_0.03_@UiO-66(Zr)-NH_2_ afforded complete conversion of nitrobenzene with 98% selectivity towards the desired *N*-phenylbenzylamine product at 90 °C under hydrogen atmosphere. Under identical conditions Au@UiO-66(Zr)-NH_2_ showed only 7% conversion of nitrobenzene with 75% selectivity to the wanted product. Similarly, Pd_0.03_@UiO-66(Zr)-NH_2_ afforded 10% conversion with 72% selectivity of the coupling product. Further, the physical mixture of Au@UiO-66(Zr)-NH_2_ and Pd_0.03_@UiO-66(Zr)-NH_2_ gave 29% conversion with 64% selectivity. These results clearly indicate that Au-Pd_0.03_@UiO-66(Zr)-NH_2_ exhibits superior activity than the related control catalysts, showing the beneficial effects of Au–Pd alloy NPs. In contrast, Au-Pd_0.03_@UiO-66(Zr) showed 29% conversion of nitrobenzene with 92% selectivity of the final product. This result suggests the importance of a suitable functionalization of the terephthalate linker in UiO-66(Zr) to boost the activity for this reaction under these conditions. Further, leaching tests by ICP-AES indicated the presence of a negligible amount of Au in the solution. Reusability test showed nearly 20% decrease of conversion after the first run and this was believed to be due to the poisoning of the alloy NPs during the reaction. Further studies are required to prove these claims with appropriate evidences.

**Figure 10:**
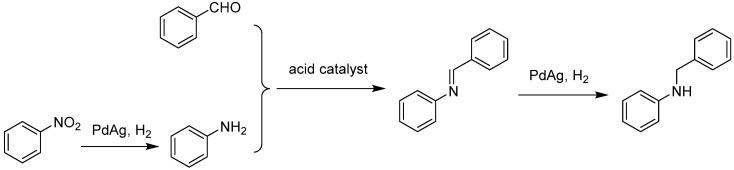
Synthesis of secondary arylamines through hydrogenation of nitrobenzene and reductive amination of benzaldehyde.

Recently, bimetallic PdAg@MIL-101(Cr) catalysts with different Pd/Ag ratios were prepared using the DSM and their activity was studied in the one-pot conversion of nitroarenes to secondary amines [52]. Powder XRD indicated that the crystallinity of MIL-101(Cr) is retained upon loading Pd/Ag species. XPS showed that both Pd and Ag atoms are in the metallic state. TEM and high-angle annular dark field scanning transmission electron microscopy (HAADF-STEM) has shown tiny PdAg NPs with the average size of 1.5 ± 0.3 nm. The activity of PdAg@MIL-101(Cr) was studied in the synthesis of secondary amines from nitroarenes via tandem reaction involving nitroarene hydrogenation, reductive amination of aldehydes or ketones, and selective hydrogenation to secondary arylamines (Figure 10). Pd@MIL-101(Cr) was efficient in rapidly completing the conversion of nitrobenzene with 56% selectivity to the wanted product after 3 h. On other hand, the alloying of Pd with Ag required a longer time to reach complete conversion, but it provided higher selectivity. Among the various catalysts tested, Pd_2_Ag_1_@MIL-101(Cr) showed complete conversion of nitrobenzene with the final 85% product selectivity. Further, the experimental results have shown that Pd NPs exhibit intrinsic hydrogenation activity, while Ag plays the role of greatly improving selectivity of the target product. On other hand, Pd/Al_2_O_3_ provided 67% conversion with 53% selectivity to the final product, which is lower than Pd@MIL-101(Cr). In addition, the physical mixture of Pd@MIL-101(Cr) and Ag@MIL-101(Cr) exhibited lower catalytic activity (70%) and lower selectivity (61%) compared to PdAg@MIL-101(Cr). This catalytic data provide indirect support for the possible formation of Pd-Ag bimetallic NPs in MIL-101(Cr). This likely possibility is, however, difficult to prove by electron microscopy analysis due to the similarity between Pd and Ag. On the other hand, commercial Pd/C exhibited comparable activity to Pd@MIL-101(Cr) catalyst, but with lower selectivity (30%), suggesting the critical role of acidity in the tandem process. The catalytic activity and selectivity of Pd_2_Ag_1_@MIL-101(Cr) was retained for three cycles, suggesting its good recyclability and durability. Powder XRD showed no loss of crystallinity after three runs. Further, no notable changes were seen for the distribution of PdAg NPs between fresh and three times used catalysts.

**Figure 11:**
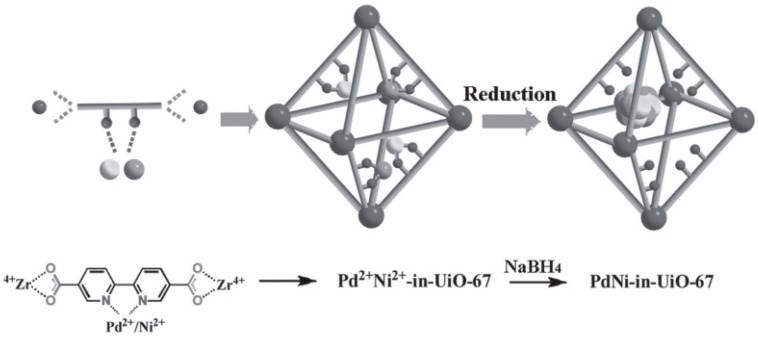
Encapsulation of PdNi NPs in UiO-67(Zr) via the in situ metal precursor incorporation method. The assembly of MOFs and incorporation of metal precursors are performed in one step. Reproduced with permission from [61].

**Figure 12:**
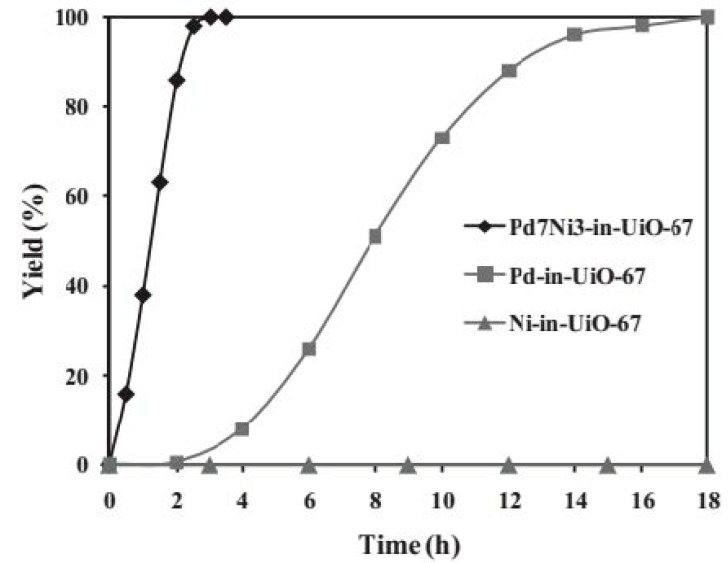
Yield of aniline versus time in the hydrogenation of nitrobenzene using Pd@UiO-67(Zr), Ni@UiO-67(Zr) and Pd_7_Ni_3_@UiO-67(Zr) catalysts. Reproduced with permission from [61].

Pd@UiO-67(Zr), Ni@UiO-67(Zr) and PdNi@UiO-67(Zr) were prepared as shown in Figure 11 and their catalytic performance was tested in the hydrogenation of nitrobenzene (Figure 12) [61]. Powder XRD confirmed that the crystalline nature of UiO-67(Zr) is retained after incorporation of these MNPs. The BET surface area values were 2212, 1801, 1683 and 1700 m^2^ g^−1^ for UiO-67(Zr), Pd@UiO-67(Zr), Ni@UiO-67(Zr), and Pd_7_Ni_3_@UiO-67(Zr), respectively. This decrease in BET surface area was taken as an indirect evidence of the internal loading of MNPs within the pores of UiO-67. TEM images indicated that the average particle size of Pd, Ni and PdNi was around 3–4 nm. XPS analysis showed the metallic state of Pd and Ni in Pd_7_Ni_3_@UiO-67(Zr). The catalytic activity of Ni@UiO-67(Zr) was studied in the hydrogenation of nitrobenzene at room temperature using hydrogen, but no activity was observed. In contrast, Pd@UiO-67(Zr) exhibited a complete conversion of nitrobenzene within 18 h. Interestingly, among the bimetallic Pd_x_Ni_y_@UiO-67(Zr) catalysts studied for this reaction under similar conditions, Pd_7_Ni_3_@UiO-67(Zr) provided the best catalytic performance affording quantitative nitrobenzene conversion within 3 h. These results were interpreted as indicating a synergistic effect in the Pd-Ni alloy as compared to monometallic MNPs. No appreciable decay in activity was observed in the five cycles of reusability test. Powder XRD of the recycled catalyst supports that the structural integrity of the MOF is mostly retained under the reaction conditions. Further, no metal leaching was observed and chemical analysis of the reused catalyst showed identical metal loading as the fresh catalyst. Furthermore, a different Pd_7_Ni_3_/UiO-67(Zr) sample was prepared following a two-step procedure consisting in the prior preparation of Pd before deposition of Ni. This different Pd_7_Ni_3_/UiO-67(Zr) exhibited an activity under identical conditions of 84% of nitrobenzene after 2 h. Besides, the activity of this Pd_7_Ni_3_/UiO-67(Zr) sample prepared in two steps dropped significantly during reusability test, showing 51% conversion in the fifth cycle. These catalytic data clearly demonstrate that the preparation procedure of MNPs within the pores of MOFs is a crucial factor to achieve optimal activity. This could be related to the statistical distribution of the different metal atoms in the NPs.

**Figure 13:**
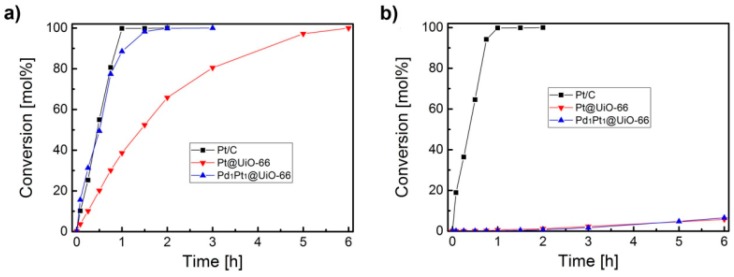
Time–conversion plot of (**a**) nitrobenzene and (**b**) 3,5-dimethylnitrobenzene using Pd_1_Pt_1_@UiO-66(Zr), Pt@UiO-66(Zr) and Pt/C as catalysts. Reproduced with permission from [62].

Preformed core–shell PdPt and RuPt NPs have been embedded into chemically robust UiO-66(Zr) MOFs and their activity was tested in the hydrogenation of nitrobenzene [62]. Powder XRD indicated the crystallinity of the UiO-66(Zr) sample upon loading these MNPs. The preformed core Pd NPs size was of 3.4 ± 0.6 nm and the addition of a Pt shell leads to an increase of the particle size. TEM images showed the average NPs sizes of 4.2 ± 0.8 nm for Pd_1_Pt_1_@UiO-66(Zr) and 4.8 ± 0.7 nm for Pd_1_Pt_2_@UiO-66(Zr). Size selective reduction of nitrobenzene and 3,5-dimethylnitrobenzene was performed with these catalysts using hydrogen as reducing agent at room temperature. No conversion of these substrates was observed with UiO-66(Zr) even after 24 h. The conversion of nitrobenzene was higher with Pd_1_Pt_1_@UiO-66(Zr) as catalyst than for Pt@UiO-66(Zr) and the value is comparable to Pt/C (Figure 13). This result indicates synergetic effects between Pd and Pt atoms in the bimetallic NPs to promote the reduction effectively. For instance, Pd_1_Pt_1_@UiO-66(Zr) was able to reduce quantitatively nitrobenzene to aniline within 2 h at room temperature.

In addition, catalysis by Pd_1_Pt_1_@UiO-66(Zr) exhibits shape selectivity. Thus, while Pt/C exhibited a similar temporal conversion profile for 3,5-dimethylnitrobenzene and nitrobenzene, Pd_1_Pt_1_@UiO-66(Zr) and Pt@UiO-66(Zr) were almost inert for 3,5-dimethylnitrobenzene reduction under identical conditions (Figure 13). Comparison of the initial reaction rate indicates that more than 99% conversion of nitrobenzene and 3,5-dimethylnitrobenzene was achieved with Pt/C featuring easy accessibility to Pt NPs located on the surface. In contrast, mono- or bimetallic NPs incorporated within UiO-66 exhibited a conversion as low as 0.2% (Pd_1_Pt_1_@UiO-66(Zr)) and 0.5% (Pt@UiO-66(Zr)) for this trisubstituted benzene. These catalytic tests strongly support the complete encapsulation of the MNPs within the UiO-66(Zr) pores as the diffusion of 3,5-dimethylnitrobenzene into the MOF voids is not feasible due to its large molecule size, thus showing the operation of size selective catalysis. Pd_1_Pt_1_@UiO-66(Zr) was reused for three cycles with no loss in its activity and size selectivity. Powder XRD measurements confirmed the structural integrity of the UiO-66(Zr) lattice after three runs, without observing any decrease in the intensity of the encapsulated MNPs. TEM images of three-times used PdPt@UiO-66(Zr) sample did not show measurable changes in the particle size.

**Figure 14:**
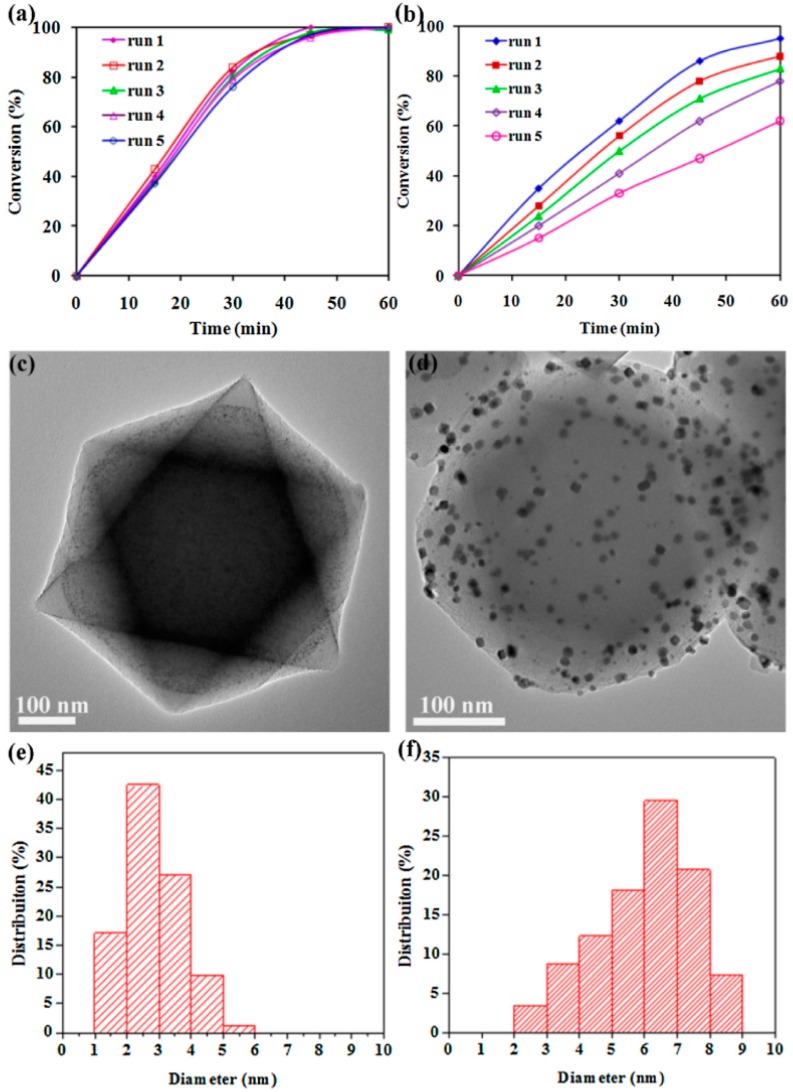
Reusability of the Pt_8_Co_1_@UiO-66(Zr) (**a**) and PtCo/UiO-66(Zr) (8:1) (**b**) in the hydrogenation of nitrobenzene. TEM images and corresponding particle-size distribution histograms of the recycled Pt_8_Co_1_@UiO-66(Zr) (**c**,**e**) and PtCo/UiO-66(Zr) (**d**,**f**) after being used five times. Reproduced with permission from [63].

Ultrafine and uniform Pt–Co alloy NPs were encapsulated within the UiO-66(Zr) pores without the assistance of any surfactant and their activity was studied in the hydrogenation of nitrobenzene [63]. Powder XRD showed that the crystal structure of UiO-66(Zr) is not affected during the loading of PtCo NPs. BET surface area values for the as-synthesized UiO-66(Zr), Pt@UiO-66(Zr), Pt_14_Co_1_@UiO-66(Zr), Pt_8_Co_1_@UiO-66(Zr), Pt_4_Co_1_@UiO-66(Zr) and Pt_1_Co_1_@UiO-66(Zr) were 1148, 930, 1011, 1043, 1055 and 1083 m^2^g^−1^, respectively. The TEM images of Pt_8_Co_8_@UiO-66(Zr) indicated homogeneous distribution of MNPs with an average size around 2 nm, suggesting the internal localization of these NPs. Further, HAADF-STEM and EDX elemental mapping confirmed that Pt and Co were evenly dispersed within the UiO-66(Zr) framework. XPS has also confirmed that Pt and Co exist in the metallic state within the pores of UiO-66(Zr). In order to demonstrate the confinement of PtCo within the pores of UiO-66(Zr), an additional catalyst containing 2 wt% PtCo catalyst deposited on the external surface of UiO-66 obtained by the impregnation method (PtCo/UiO-66(Zr)) was also prepared. TEM images of PtCo/UiO-66(Zr) revealed that most of NPs in this sample are located on the external surface with large particle size around 7–8 nm. It was proposed that the difference in particle size distribution between Pt_1_Co_1_@UiO-66(Zr) and PtCo/UiO-66(Zr) reflects the effect of the MOF structure impeding particle growth. The activity of these two solids was tested in the hydrogenation of nitrobenzene using hydrogen as reducing agent. Pt_8_Co_1_@UiO-66(Zr) exhibited the highest catalytic activity, providing quantitative conversion under atmospheric hydrogen pressure at 25 °C within 45 min. On other hand, under identical conditions, Pt@UiO-66(Zr) reached 85% conversion after 60 min. These results indicate that Pt_8_Co_1_@UiO-66(Zr) outperforms Pt@UiO-66(Zr). This superior activity of Pt_8_Co_1_@UiO-66(Zr) was attributed to the effect of Co doping. On other hand, PtCo/UiO-66(Zr) (with 8:1 Pt/Co) gave remarkably inferior activity in the hydrogenation of nitrobenzene thus, showing the benefits of the confinement of the NPs to achieve small particle size. Reusability experiments did not reveal a significant loss in the activity up to five cycles (Figure 14). Further, powder XRD and TEM measurements indicated no changes in crystallinity and particle size, respectively (Figure 14). In contrast, PtCo/UiO-66 showed poor reusability due to the severe aggregation of NPs under reaction conditions (Figure 14).

**Figure 15:**
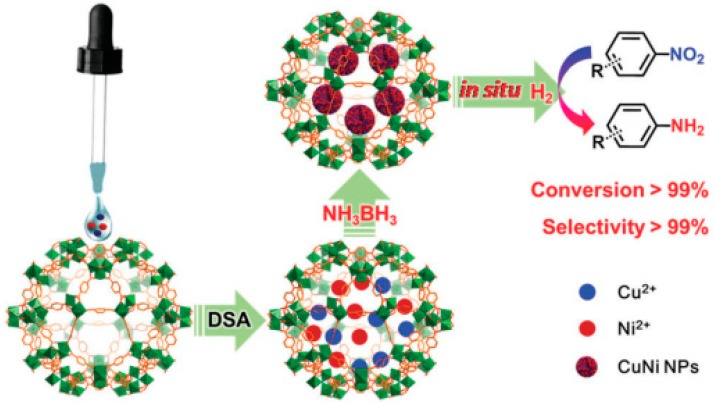
Preparation procedure of CuNi@MIL-101(Cr) by the DSM and its use as catalyst for a cascade reaction involving dehydrogenation of NH_3_BH_3_ and hydrogenation of nitroarenes. Reproduced with permission from [64].

Bimetallic CuNi NPs were confined inside MIL-101(Cr) to obtain CuNi@MIL-101(Cr) using DSM and their catalytic performance was investigated in the cascade reactions of NH_3_BH_3_ dehydrogenation and nitroarene reduction under mild conditions (Figure 15) [64]. Powder XRD indicated no loss of crystallinity upon loading CuNi NPs. TEM images revealed that CuNi NPs exist with the particle size around 3 nm and the HRTEM image with a lattice fringe distance of 0.206 nm indicates that the CuNi NPs form an alloy. Further, Cu and Ni loading was found to be 1.09 wt% and 2.65 wt%, respectively as determined by ICP-AES. The BET surface areas of MIL-101(Cr) and CuNi@MIL-101(Cr) were 3660 and 1983 m^2^g^−1^, respectively. Interestingly, the TOF achieved for the hydrogenation of nitrobenzene using CuNi@MIL-101(Cr) at room temperature was around 99 mol_nitrobenzene_ mol_Ni_^−1^ h^−1^ which is much better than those cascade reactions reported on the use of MNPs@MOFs with noble metals [52,84,85,86]. Various nitroarenes with electron-donating substituents were reduced to their respective amines under identical conditions. In contrast, the aniline yield was around 2% after 19 h with hydrogen as reducing agent, indicating that the limited hydrogen gas dissolved in the solution significantly affected the rate of the reaction. The activity of CuNi@MIL-101(Cr) was maintained for twenty consecutive cycles without any activation treatment, thus showing superior nature of the catalyst. Further, the size and morphology of CuNi NPs were also not affected after the twenty runs, thus showing the robust nature of these NPs within the pores of MIL-101(Cr). In contrast, CuNi/MIL-101(Cr) prepared by wet impregnation maintained its activity for four consecutive cycles and its activity gradually decreased to 85, 81 and 79% for the subsequent 5^th^–7^th^ cycles, respectively. This gradual activity loss was due to the lack of stability of CuNi NPs located outside of MIL-101(Cr) pores.

**Figure 16:**
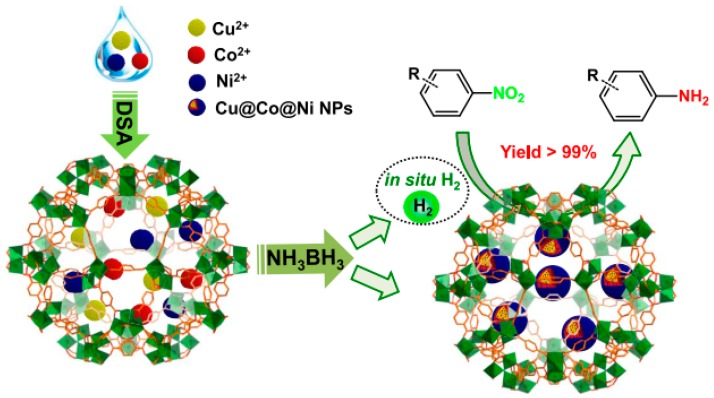
Preparation procedure for Cu@Co@Ni/MIL-101(Cr) and its use as catalyst for a cascade reaction involving the dehydrogenation of NH_3_BH_3_ and subsequent hydrogenation of nitroarenes. Reproduced with permission from [65].

Tiny Cu@Co@Ni core–shell NPs comprising of Cu core, Co middle shell and Ni outer shell stabilized inside MIL-101(Cr) MOF (Cu@Co@Ni/MIL-101(Cr)) were synthesized and their catalytic activity tested for the in situ hydrogenation of nitroarenes using hydrogen generated by the decomposition of NH_3_BH_3_ under mild conditions (Figure 16) [65]. TEM images of Cu@Co@Ni/MIL-101(Cr) indicated a uniform dispersion of Cu@Co@Ni NPs with the average particle size around 3.3 nm (Cu/Co/Ni molar ratio of 0.33:0.33:0.33). BET surface area measurements indicated that the as-synthesized MIL-101(Cr) and Cu@Co@Ni/MIL-101(Cr) have values of 3425 and 2148 m^2^ g^−1^, respectively. XPS analysis revealed the presence of zero-valent copper, cobalt and nickel in Cu@Co@Ni/MIL-101(Cr) catalyst. Among the various catalysts (monometallic Cu, Co, and Ni; bimetallic Cu@Co and Cu@Ni; and trimetallic CuCoNi alloy NPs) compared for the decomposition of NH_3_BH_3_ to hydrogen, experimental data showed that Cu@Co@Ni exhibits the highest activity, releasing the full theoretical amount of hydrogen in 14 min. In contrast, an analogous catalyst with identical metal loading and NP size, namely CuCoNi/ZIF-8 required 30 min for the decomposition of NH_3_BH_3_ under similar experimental conditions, which is much longer than the time measured for Cu@Co@Ni/MIL-101(Cr). This superior activity of Cu@Co@Ni/MIL-101(Cr) is due to the high population of active sites without the drastic diffusion limitations occurring in ZIF-8. Interestingly, the Cu@Co@Ni/MIL-101(Cr) completed the reaction within 10 min at 25 °C and 5.5 min at 30 °C with TOF values of 31 and 56 mol_H2_ molcat^−1^ min^−1^ which are comparatively higher than the TOF values reached by other catalysts including those based on noble metal catalysts [87,88,89,90]. Later, the catalytic performance of Cu@Co@Ni/MIL-101(Cr) was examined in the cascade reaction of NH_3_BH_3_ decomposition to hydrogen and subsequent reduction of nitrobenzene. Conversion of nitrobenzene to aniline with 99% yield was achieved using Cu@Co@Ni/MIL-101(Cr) as catalyst within 5 min at 20 °C. The scope of this catalyst was further expanded to a series of diverse substituted aromatic nitroarenes with electron donating and electron-withdrawing substituents affording in most of the cases > 99% yields within 5 min at 20 °C. Reusability tests under similar experimental conditions showed identical catalytic performance and maintenance of the framework integrity for Cu@Co@Ni/MIL-101(Cr) after five consecutive uses. Furthermore, TEM images of the reused catalyst did not show any growth of the particle size, thus supporting catalyst stability under the experimental conditions.

## 4. Summary and Outlook

The present review has shown that the incorporation of MNPs inside structurally robust MOFs is a general methodology to obtain highly efficient and frequently stable heterogeneous catalysts to promote the reduction of nitro groups. Depending on the reducing agent, different transition metals should be used as catalysts for this reaction as described in the review. Besides the transformation of nitro to amino group, it has also been shown that MNPs inside MOFs can behave as multifunctional catalysts that can promote tandem reactions in where more than one elementary step is combined in a single process promoted by same catalyst. Tandem reactions represent a clear advantage of process intensification avoiding intermediate workup and separation processes.

It has also been shown that there are possibilities to prepare nano alloys of more than one metal inside MOF cavities. Bimetallic and trimetallic catalysts provide sites with unique catalytic activity different from that of analogous catalysts having a single metal. The electronic density and the generation of partial charges can play a remarkable role in catalysis by MNPs and these parameters can be tuned by forming alloys with the appropriate composition and morphology. In this regard, it can be expected that the number of studies dealing with encapsulated alloys will grow in the near future, including the influence of morphology either as core–shell or uniform atomic distribution.

It is also a current tendency to increase the number of studies in which the active sites of the MNPs cooperate the activity of the MOFs. It is therefore expected that the field will continue growing with further examples of different MOFs including two-dimensional and mixed-metal MOFs in the near future.

## Figures and Tables

**Table 1:** List of various MNPs@MOFs used as heterogeneous solid catalysts for reduction of nitrobenzene.

Catalyst	MNPs (Size, nm)	Reducing Agent	Catalytic Reaction	No. Reuses	Ref.
Pd@MIL-101(Cr)	Pd (2.5 ± 0.3)	H_2_	Synthesis of 2-(4-aminophenyl)-1*H*-benzimidazole from 4-nitrobenzaldehyde	3	[52]
Pd-DUT-67	Pd (3.5)	H_2_	Hydrogenation of nitrobenzene	-	[53]
PdC_60_@UiO-67(Zr)	Pd (5 ± 2)	H_2_	Synthesis of *N*-benzylaniline from nitrobenzene	5	[54]
Ru-UiO-66(Zr)	Ru (1.07)	HCOOH	Hydrogenation of nitrobenzene	6	[55]
Au@MOF-3	Au (1.85 ± 0.83)	NaBH_4_	4-Nitrophenol reduction	5	[56]
Au@ZIF-8(Zn,Cu)	Au nanoclusters (<2)	NaBH_4_	4-Nitrophenol reduction	10	[57]
Fe_3_O_4_@MIL-100(Fe)-Pt	Pt (1.9 ± 0.2)	NaBH_4_	4-Nitrophenol reduction	10	[58]
Ni@MesMOF-1	Ni (1.4)	NaBH_4_	Hydrogenation of nitrobenzene	3	[59]
AuPd@UiO-66(Zr)-NH_2_	Au-Pd_0.03_ (5.3)	H_2_	Reductive amination of nitrobenzene	5	[60]
PdAg@MIL-101(Cr)	PdAg (1.5 ± 0.3)	H_2_	Synthesis of secondary arylamines by hydrogenation of nitrobenzene	3	[52]
Pd_7_Ni_3_@UiO-67(Zr)	PdNi (3–4)	H_2_	Hydrogenation of nitrobenzene	5	[61]
Pd_1_Pt_1_@UiO-66(Zr)	PdPt (4.2 ± 0.8)	H_2_	Hydrogenation of nitrobenzene	3	[62]
Pt_8_Co_1_@UiO-66(Zr)	PtCo (2)	H_2_	Hydrogenation of nitrobenzene	5	[63]
CuNi@MIL-101(Cr)	CuNi (3)	H_2_	Hydrogenation of nitrobenzene	7	[64]
Cu@Co@Ni/MIL-101(Cr)	Cu@Co@Ni (3.3)	H_2_	Hydrogenation of nitrobenzene	5	[65]
